# Prevention of hypokalaemia in bulimia nervosa

**DOI:** 10.1186/2050-2974-3-S1-O61

**Published:** 2015-11-23

**Authors:** Leanne Barron, Warren Ward

**Affiliations:** 1 QUT Eating Disorder Clinic, Brisbane, Australia; 2 Brisbane City Doctors, Brisbane, Australia; 3 Royal Brisbane Women's Hospital, Brisbane, Australia

## 

Hypokalaemia represents a significant risk factor for sudden cardiac death in patients with bulimia nervosa. Whilst the goal of treatment is always reduction of vomiting, this may not be easily attainable and in the interim some patients may be at significant risk.

As a harm minimisation strategy, it was suggested that Nexium (esomeprazole) could reduce hypokalemia. This is a logical manoeuvre in view of the mechanism for potassium depletion being renal compensation for hydrogen loss in the vomitus.

After discussion with their treating psychiatrist, two patients who had required intensive potassium repletion (including via ICU and PIC lines) were commenced on a low dose of Nexium as outpatients. ECGs were monitored (particularly as Nexium may prolong the QT interval). Potassium supplements were continued, and electrolytes were monitored daily initially.

Despite both patients continuing to binge/purge at similar frequency, their potassium levels remained at levels in the low end of normal range. Neither patient required intravenous potassium, although levels did fall intermittently (when patients omitted doses of Nexium). Both commented that they felt they “didn't deserve” such an effective treatment, but eventually their compliance improved. Unfortunately one patient later developed QT prolongation and the nexium had to be discontinued. However, commencement of ranitidine is currently underway on the assumption that this will similarly reduce hydrogen and hence potassium loss. Nexium is known to cause prolonged QT, and this is a risk for which patients would need to be monitored medically with regular ECGs. It remains to be seen whether ranitidine is similarly effective in preventing hypokalaemia. If this is indeed the case it may prove a safer option.

